# Cytotoxic Phenylpropanoids and a New Triterpene, Turformosinic Acid, from *Turpinia formosana* Nakai

**DOI:** 10.3390/molecules17021837

**Published:** 2012-02-14

**Authors:** Hui-Chi Huang, Chun-Tang Chiou, Ping-Chun Hsiao, Chia-Ching Liaw, Li-Jie Zhang, Chao-Lin Chang, Ih-Sheng Chen, Wen-Chi Chen, Kuo-Hsiung Lee, Yao-Haur Kuo

**Affiliations:** 1 School of Chinese Pharmaceutical Sciences and Chinese Medicine Resources, China Medical University, Taichung 404, Taiwan; 2 Division of Herbal Drug and Natural Products, National Research Institute of Chinese Medicine, Taipei 112, Taiwan; 3 College of Pharmacy, Kaohsiung Medical University, Kaohsiung 807, Taiwan; 4 Graduate Institute of Integrated Medicine, College of Chinese Medicine, China Medical University, Taichung 404, Taiwan; 5 Natural Products Research Laboratories, UNC Eshelman School of Pharmacy, University of North Carolina, Chapel Hill, NC 27599, USA; 6 Chinese Medicine Research and Development Center, China Medical University and Hospital, Taichung 404, Taiwan

**Keywords:** Staphyleaceae, *Turpinia formosana* Nakai, turformosin A, turfomosinic acid, cytotoxicity

## Abstract

One new phenylpropanoid, turformosin A (**1**), and one new triterpene, turformosinic acid (**2**), together with 16 known compounds, were isolated from the stems of *Turpinia formosana* Nakai. All structures were elucidated on the basis of spectroscopic analysis, including 1D- and 2D-NMR techniques and MS analysis. Selected isolated compounds were evaluated for *in vitro* cytotoxicity against four human cancer cell lines and antioxidant scavenging effects on DPPH. (−)-(7′*S*,8′*S*)-*threo*-carolignan X (**3**) exhibited cytotoxicity against Hep2, WiDr, Daoy, and MCF-7 cell lines with ED_50_ values of 3.60, 4.45, 6.07, and 13.7 μg/mL, respectively. Turformosin A (**1**), (−)-(7′*S*,8′*S*)-*threo*-carolignan X (**3**), methoxyhydroquinone-4-β-D-glucopyranoside (**5**), and methoxy-hydroquinone-1-β-D-glucopyranoside (**6**), exhibited similar anti-oxidative activity. Hep2 cells treated with 10 μg/mL of **3** showed elevation of sub-G1 population (from 20% at 8 h to 60% at 48 h), and activation of caspase-9/caspase-3/PARP cascade. Compound **3** induced intrinsic apoptotic pathway in Hep2 cells with dose and time dependence (10 μg/mL for 8 h).

## 1. Introduction

*Turpinia formosana* Nakai (Staphyleaceae) is an endemic plant which is widely found in the southern Taiwanese mountains. The roots have been used in Taiwanese traditional medicine to activate blood circulation, reduce swelling, and relieve pain and splenomegaly [1]. The genus *Turpinia* contains ten species found in subtropical and tropical Asia and America [2]. In previous pharmaceutical investigations on crude extracts of *Turpinia *sp., *T. pomifera* showed antimalarial activity against *Plasmodium falciparum *[3], and *T. ternate* demonstrated cytotoxicity against *Artemia salina *larvae as well as antioxidant activity [4]. Other Staphyleaceous plants, including *Staphylea pinnata* and *Stephanandra tanakae*, exhibited anti-bacterial [5] and cytotoxic [6] effects, respectively. Previous phytochemical studies showed that plants from this family contain ellegic acid derivatives [4], megastigmans [7], megastigman glycosides [8,9,10], flavonol glycosides, anthocyanins [11], amino acid amides, and triterpenoic acids [12].

**Figure 1:**
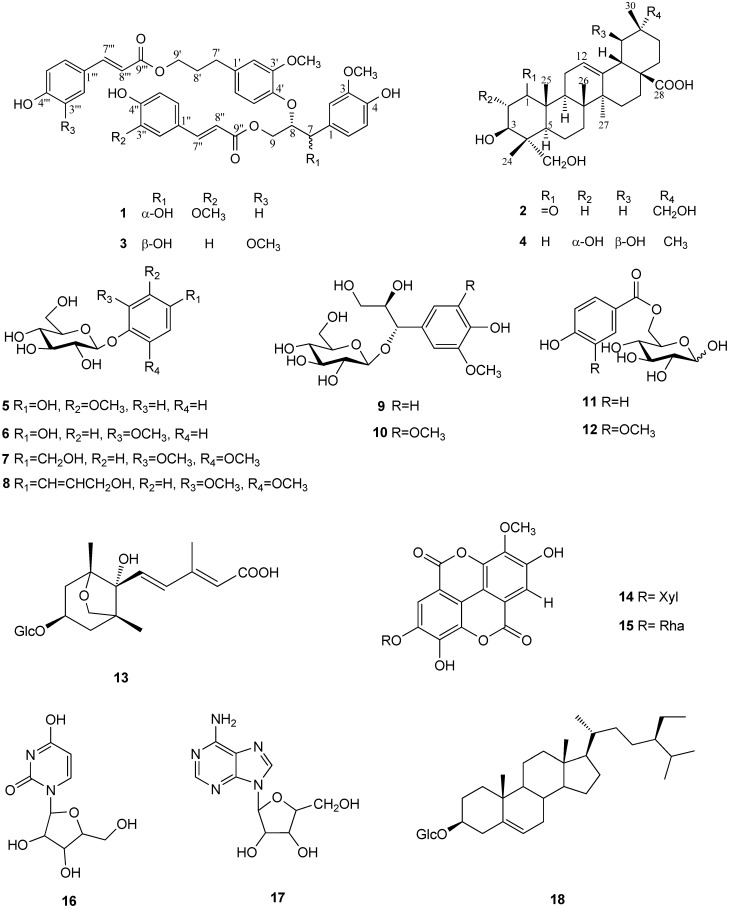
Chemical structure of compounds **1**–**18** from *T. formosana* Nakai.

In our continuing search for anticancer principles from Taiwanese plants, we found that an EtOH extract of the stems of *T. formosana* Nakai showed cytotoxic activity against the Hep2 and Daoy cell lines. However, no detailed reports are available concerning the chemistry and pharmacological activities of this plant. Our subsequent phytochemical analysis of the EtOH extract has led to the isolation of a new phenylpropanoid, turformosin A (**1**), and a new oleanane-type triterpene, turformosinic acid (**2**), as well as 16 known compounds (**3**–**18**) (Figure 1).

The structures of the two new compounds were elucidated by extensive spectroscopic methods including 1D- and 2D-NMR experiments. Selected isolated compounds (**1**–**6** and **9**–**16**) were also evaluated for cytotoxic and antioxidative activities. (−)-(7′*S*,8′*S*)-*threo*-Carolignan X (**3**) [13] showed strong cytotoxicity against Hep2 cells. In addition, this is the first report on the cytotoxic activities of (−)-(7′*S*,8′*S*)-*threo*-carolignan X (**3**) and induced apoptosis in Hep2 cells is also described. 

## 2. Results and Discussion

### 2.1. Structure Determination

**Figure 2:**
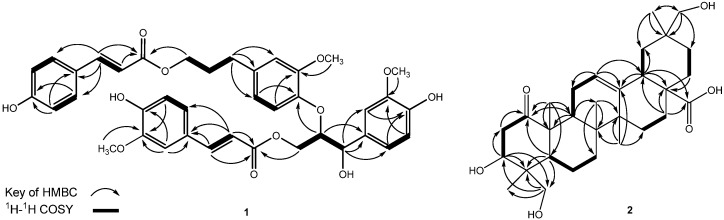
Key HMBC (arrow) and ^1^H-^1^H COSY (bold lines) correlations of compounds **1**–**2**.

Compound **1** was isolated as a colorless amorphous solid. A sodiated ion peak at *m/z *723.2420 ([M+Na]^+^; calc. 723.2417) in the HR-ESI-MS of **1** was consistent with the molecular formula C_39_H_40_O_12_. The UV absorption maxima (λ_max_ = 229, 289, and 317 nm) indicated the presence of aromatic moieties. The IR absorption bands of **1** were characteristic for hydroxy (ν_max_ = 3,398 cm^−1^), carbonyl (ν_max_ = 1,687 cm^−1^), and aromatic ring (ν_max_ = 1,603 and 1,508 cm^−1^) moieties. The ^1^H-NMR spectrum of **1** (Table 1) showed four sets of phenyl signals with one A_2_B_2_ and three ABX coupled systems at δ_H_ 6.64 ~ 7.32. One ABX set of aromatic signals at δ_H_ 6.76 (H-5), 6.88 (H-6), and 7.07 (H-2), together with three oxygenated protons at δ_H_ 4.45 (2H, m, H-9), 4.60 (1H, m, H-8), 4.92 (1H, d, *J* = 5.5 Hz, H-7), as well as a methoxy group [δ_H_ 3.79 (3H, s, OCH_3_)], suggested the presence of a (4-hydroxy-3-methoxyphenyl)-7,8,9-propantriol moiety in the molecule [14]. A second ABX-type coupling system consisted of three aromatic proton signals at δ_H_ 6.64 (H-5'), 6.73 (H-2') and 6.84 (H-6'). Based on HMBC correlations (Figure 2), this phenyl ring was substituted with a methoxy group [δ_H_ 3.72 (s)] and a propanol unit [δ_H_ 1.89 (2H, t, *J *= 7.0 Hz, H-8'), 2.59 (2H, dd, *J *= 7.5, 7.0 Hz, H-7'), and 4.09 (2H, d, *J* = 6.0 Hz, H-9')]. Thus, the second aromatic unit was established as a (4-hydroxy-3-methoxyphenyl)-9'-propanol system [14]. A third phenylpropanoid unit was assigned as a feruloyl unit, based on the presence of characteristic signals for an aromatic moiety with an ABX pattern system [δ_H_ 7.13 (1H, s, H-2''), 7.02 (1H, d, *J* = 8.0 Hz, H-6'') and 6.80 (1H, d, *J *= 8.0, H-5'')], a methoxy group (δ_H_ 3.84), a pair of *trans*-vinyl groups [δ_H_ = 7.56 (1H, d, *J* = 16.0, H-7'') and 6.32 (1H, d, *J* = 16.0, H-8'')], and an ester carbonyl carbon [δ_C_ 169.3 (C-9'')] [15]. The remaining aromatic signals showed an A_2_B_2_ coupling pattern [δ_H_ 7.32 (2H, d, *J* = 8.0 Hz, H-2''' and 6''') and 6.78 (2H, m, H-3''' and 5''')] linked to a pair of *trans*-vinyl protons [δ_H_ 7.36 (1H, d, *J* = 16.0 Hz, H-7''') and 6.14 (1H, d, *J *= 16.0 Hz, H-8''')], and an ester carbonyl carbon [δ_C_ 168.9 (C-9''')]. The fourth aromatic system was therefore identified as a 4'''-hydroxycinnamoyl unit. 

**Table 1:** ^1^H-NMR and ^13^C-NMR spectroscopic data (*δ* in ppm, *J* in Hz) of compound **1**.

				1				
Position	*δ*_H_^a^	*δ*_C_^b^	*δ*_C_^c^		Position	*δ*_H_^a^	*δ*_C_^b^	*δ*_C_^c^
1	-	133.8	131.0		1''	-	127.6	126.7
2	7.07 (s)	111.6	108.9		2''	7.13 (s)	111.7	109.4
3	-	148.7	146.8		3''	-	150.5	146.6
4	-	146.9	145.1		4''	-	149.2	148.1
5	6.76 (d, *J* = 8.0)	115.8	114.2		5''	6.80 (d, *J* = 8.0)	116.4	115.0
6	6.88 (d, *J* = 8.0)	120.8	119.3		6''	7.02 (d, *J* = 8.0)	124.1	123.1
7	4.92 (d, *J* = 5.5)	74.1	72.3		7''	7.56 (d, *J* = 16.0)	146.6	145.2
8	4.60 (m)	83.7	84.2		8''	6.32 (d, *J* = 16.0)	115.5	115.1
9	4.45 (m)	64.8	62.8		9''	-	169.3	167.6
MeO-3	3.79 (s)	56.4	55.8		MeO-3''	3.84 (s)	56.4	55.9
1'	-	137.5	137.2		1'''	-	127.0	126.5
2'	6.73 (s)	113.9	112.4		2'''	7.32 (d, *J* = 8.0)	131.2	130.0
3'	-	151.9	151.1		3'''	6.78 (m)	116.8	115.9
4'	-	147.2	145.0		4'''	-	161.1	158.4
5'	6.64 (d, *J* = 7.5)	121.7	120.4		5'''	6.78 (m)	116.8	115.9
6'	6.84 (d, *J* = 7.5)	119.7	121.1		6'''	7.32 (d, *J* = 8.0)	131.2	130.0
7'	2.59 (dd, *J* = 7.5, 7.0)	32.8	31.9		7'''	7.36 (d, *J* = 16.0)	146.7	145.2
8'	1.89 (t, *J* = 7.0)	31.4	30.3		8'''	6.14 (d, *J* = 16.0)	114.9	114.5
9'	4.09 (t, *J* = 6.0)	64.8	63.8		9'''	-	168.9	164.4
OMe-3'	3.72 (s)	56.4	55.8					

^a^ 500 MHz for ^1^H-NMR in CD_3_OD; ^b^ 125 MHz for ^1^^3^C-NMR in CD_3_OD; ^c^ 125 MHz for ^1^^3^C-NMR in CDCl_3_.

Moreover, the assignments for the four phenylpropanoid units based on the above proton NMR data were also supported by appropriate carbon signals in the ^13^C-NMR (Table 1) and HMQC spectra of **1**. 

Compound **1** is thus closely related to (−)-(7′*S*,8′*S*)-*threo*-carolignan X (**3**), except for the position of feruloyl and 4-hydroxycinnamoyl units and the stereochemistry of the 7,8,9-propantriol moiety [13]. Inspection of the HMBC spectrum of **1** revealed cross peaks between the following proton and carbon signals: H-8 (δ_H_ 4.60) with C-4' (phenoxy ring δ_C_ 147.2), H-9 (δ_H_ 4.45) with C-9'' (δ_C_ 169.3, carbonyl carbon in ferulic acid), and H-9' (δ_H_ 4.09) with C-9''' (δ_C_ 168.9, cinnamic acid carbonyl), which determined the planar structure of **1** to be 7-(4-hydroxy-3-methoxyphenyl)-9-(4-hydroxy-3-methoxy-cinnamoyloxy)-8{4'-[9'-(4-hydroxycinnamoyloxy)-7'-propenyl]-3'-methoxyphenoxy}-1-propanol. Due to the presence of two chiral carbons in the 7,8,9-trioxygenated propane moiety of **1**, two possible diastereomers (*erythro *and *threo*) are possible for **1**. Comparison of the chemical shift and coupling constant data of H-7 and H-8 with **3** and **1** indicated that **1** belongs to the *erythro* series [15,16]. When the ^1^H-NMR spectrum of **1** and **3** was measured in CDCl_3_ instead of CD_3_OD, the coupling constants for H-7 were clearly observed. Therefore, we also use the CDCl_3_ to analyze the ^1^H- and ^13^C-NMR of **1** and **3**. The proton resonance of H-7 in compound **3** appeared as a doublet and higher coupling constant (*J*_H7-H8_ = 8.0 Hz) determined the *threo* diastereomer, which was further deduced to possess a *trans* diaxial orientation. As to compound **1**, the H-7 proton resonance had a small coupling constant (*J*_H7-H8_ = 3.2 Hz), indicating the presence of the *erythro* diastereomer. Comparison of the ^13^C spectra (CDCl_3_) of **1** and **3**, compound **1** has higher field C-7 signals (δ_C_ 72.3 in **1**; δ_C_ 74.4 in **3**), C-8 (δ_C_ 84.2 in **1**; δ_C_ 86.2 in **3**), and C-9 (δ_C_ 62.8 in **1**; δ_C_ 63.1 in **3**) revealing the *erythro* form of **1**, rather than the *threo* form of **3** [13]. Therefore, *erythro*-**1** have the (7*R*,8*S*) configuration and *threo*-**3** would be 7S,8S configuration. Based on the above data, **1** was elucidated as *erythro*-7-(4-hydroxy-3-methoxyphenyl)-9-(4-hydroxy-3-methoxycinnamoyloxy)-8{4'-[9'-(4-hydroxycinnamoyloxy)-7'-propenyl]-3'-methoxyphenoxy}-1-propanol and has been named as turformosin A.

Compound **2** was obtained as a colorless amorphous solid. A molecular ion peak at *m/z* 502.3290 [M]^+^ in the HR-ESI-MS spectrum suggested its molecular formula to be C_30_H_46_O_6_ (calculated mass: *m/z* 502.3294). The ^1^H-NMR spectrum (Table 2) revealed the presence of five methyl groups [δ_H_ = 1.33, 1.19, 0.92, 0.87, and 0.86 (each 3H, s)], two hydroxymethylene groups at δ_H_ 3.48 and 3.33 (each 1H, d, *J* = 11.2 Hz), 3.19 (2H, br s), one olefinic proton [δ_H_ 5.24 (1H, t-like, *J* = 4.0 Hz)], and an oxygen-bearing methine at δ_H_ 3.81 (1H, dd, *J* = 12.0, 4.8 Hz). The ^13^C-NMR (Table 2) and DEPT spectra of **2** showed 30 carbon signals including five methyls, eleven methylenes, five methines, seven quaternary carbons and two carbonyl carbons. Signals for olefinic proton at δ_H_ 5.24 and two olefinic carbons at δ_H_ 124.0 (C-12) and 144.4 (C-13), are indicative of an olen-12-en [17]. The ^13^C NMR data for rings B-D of **2** were similar to those of **4**. However, the ^13^C NMR spectrum of **2** showed the presence of signals for a carbonyl moiety (δ_C_ 215.0). The carbonyl group was assigned at C-1 (δ_C_ 215.0), resulting in a deshielding effect on C-2 and C-10 (*ca.* δ_C_ 25.0 and 14.0, respectively), as compared to **4**.

**Table 2:** ^1^H-NMR and ^13^C-NMR spectroscopic data^a^ (*δ* in ppm, *J* in Hz) of compound **2** in CD_3_OD.

	2					
Position	δ_H_^b^	δ_C_^c^		Position	δ_H_^b^	δ_C_^c^
1	-	215.0		16	1.99 (dd, *J* = 13.2, 3.2)1.60 (m)	24.0
2	2.29 (dd, *J* = 12.0, 5.2)3.10 (t, *J* = 12.0)	44.6		17	-	48.0
3	3.81 (dd, *J* = 12.0, 4.8)	73.3		18	2.88 (dd, *J* = 13.6, 4.0)	42.1
4	-	44.0		19	1.80 (m), 1.09 (m)	41.0
5	1.35 (m)	47.6		20	-	36.7
6	1.56 (m)	18.4		21	1.25 (d, *J* = 8.8)1.76 (m)	33.0
7	1.27 (dd, *J* = 10.0, 3.2)1.58 m	33.2		22	1.14 (m)1.47 (m)	29.2
8	-	40.3		23	3.48 (d, *J* = 11.2)3.33 (d, *J* = 11.2)	65.8
9	2.25 (dd, *J* = 10.4, 5.6)	40.2		24	0.86 (s)	13.2
10	-	53.2		25	1.33 (s)	15.9
11	2.27 (m), 1.83 (m)	26.2		26	0.87 (s)	18.3
12	5.24 (t-like, *J* = 4.0)	124.0		27	1.19 (s)	26.3
13	-	144.4		28	-	181.7
14	-	43.1		29	3.19 (br s)	74.3
15	1.74 (m), 1.06 (m)	28.7		30	0.92 (s)	19.5

^a^ Assignments made using the HSQC and HMBC techniques; ^b^ 400 MHz for ^1^H-NMR; ^c^ 100 MHz for ^13^C-NMR.

The HMBC spectrum (Figure 2) of **2** showed correlations between the oxymethine proton signal at δ_H_ 3.81 (H-3) and the carbon signals at δ_C_ 44.6 (C-2), 44.0 (C-4), and 13.2 (C-24). From the coupling constant of H-3 (*J *= 12.0, 4.8), the hydroxy group at C-3 was assigned as having a β-orientation [18]. 

HMBC correlations between the proton at δ_H_= 3.48 and carbons at δ_C_ 13.2 (C-24), 44.0 (C-4), and 47.6 (C-5), between δ_H_ 3.33 and δ_C_ 13.2 (C-24) and 44.0 (C-4), and between δ_H_3.19 and δ_c_41.0 (C-19), 36.7 (C-20), 33.0 (C-21) and 19.5 (C-30), suggested that the two secondary hydroxy groups were connected to C-23 and C-29, respectively. 

**Figure 3:**
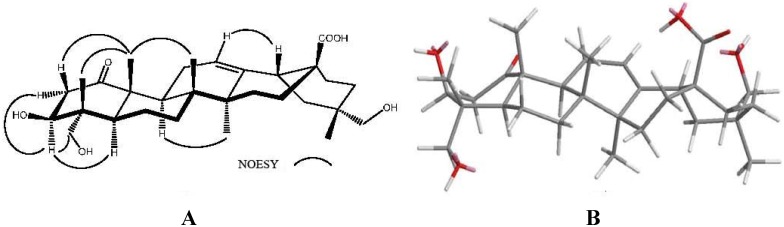
(**A**) Key NOESY (curve) correlations of compound **2**; (**B**) Computer-generated perspective model of **2** using MM2 force field calculation.

The cross-peaks between C-1 (δ_C_ 215.0) and H-2a (δ_H_ 2.29), H-2b (δ_H_ 3.10), and Me-25 (δ_H_ 1.33) in the HMBC spectrum also agreed with this assignment. The complete ^1^H- and ^13^C-NMR spectroscopic assignments were established by the analyses of ^1^H-^1^H COSY, HMQC and HMBC data. The relative stereochemistry was determined by its MM2-minimized energy-calculated molecular model drawing with NOESY correlations (Figure 3). The NOESY spectrum showed the correlations of Me-25/H-2b, Me-24 and Me-26, H-3/H-2a, H-5 and H_2_-23, as well as H-9/H-5 and Me-27, suggesting that 3-OH, Me-24, Me-25, and Me-26 were all in β-orientation. On the basis of the above evidence, the structure of **2** was established as 3β,23,29-trihydroxy-1-oxo-olean-12-en-28-oic acid, and **2** has been given the name turformosinic acid.

The 16 additional isolated compounds were identified as (−)-(7′*S*,8′*S*)-*threo*-carolignan X (**3**) [13], 2α,3β,19β-23-tetrahydroxyolean-12-en-28-oic acid (**4**) [17], methoxyhydroquinone-4-β-D-glucopyranoside (**5**) [19], methoxyhydroquinone-1-β-D-glucopyranoside (**6**) [20], 3,5-dimethoxybenzyl alcohol 4-*O*-β-D-glucopyranoside (**7**) [21], 4-(3-hydroxy-1-propenyl)-2,6-dimethoxyphenyl β-D-glucopyranoside (**8**) [22], d-*threo*-guaiacylglycerol-*O*-β-D-glucopyranoside (**9**) [23], (7*S*,8*R*)-syringoylglycerol-7-*O*-β-D-glucopyranoside (**10**) [24], 6-*O*-(*p*-hydroxybenzoyl)-D-glucose (**11**) [25], d-glucose-6(4-hydroxy-3-methoxybenzoate) (**12**) [26], dihydrophaseic acid 4'-*O*-β-D-glucopyranoside (**13**) [27], 3'-*O*-methyl ellagic acid 4-*O*-β-D-xylopyranoside (**14**) [28], 3'-*O*-methyl ellagic acid 4-*O*-α-L-rhamnopyranoside (**15**) [29], uridine (**16**) [30], adenosine (**17**) [31], and β-sitosteryl glucoside (**18**) [32], respectively, by comparison with the spectroscopic data reported in the literature for these compounds. 

### 2.2. Cytotoxicity and DHHP Radical Scavenging Activity

Most of isolated compounds (**1**–**6** and **9**–**16**; >1.0 mg), except for compounds **7** and **8** due to their limited amounts (<1.0 mg), were evaluated for cytotoxicity against human tumor cell lines (Hep2, WiDr, Daoy, and MCF-7) and antioxidant scavenging effects on DPPH (Table 3), using mitomycin C and α-tocopherol as positive controls, respectively. Compound **3** showed ED_50_ values of 3.60, 4.45, 6.07, and 13.69 μg/mL against Hep2, WiDr, Daoy, and MCF-7 cell lines, respectively, whereas **1** exhibited much lower ED_50_ values of 13.22, 12.07, and 11.46 μg/mL against the Hep2, WiDr, and Daoy cell lines, respectively. In addition, **1**, **3**, **5**, and **6** exhibited significant antioxidant effects with IC_50_ values of 26.9, 28.4, 23.6, and 30.8 μg/mL, respectively. Compounds **2**, **4**, and **9**–**16** showed neither cytotoxic nor anti-oxidative activity.

**Table 3:** Cytotoxicity and antioxidant scavenging effects of compounds **1**–**6** and **9**–**16**.

**Compound**	****	**Anti-oxidative ^a^**		**Cytotoxicity ^b^**			
		DPPH test		MCF-7	Daoy	WiDr	Hep2
		ED_50_ (μg/mL)		ED_50_ (μg/mL)			
**1**		26.9		- ^c^	11.46	12.07	13.22
**3**		28.4		13.69	6.07	4.45	3.60
**5**		23.6		-	-	-	-
**6**		30.8		-	-	-	-
Positive control		12.25 ^d^		0.15 ^e^	0.08 ^e^	0.15 ^e^	0.16 ^e^

^a^ Compounds **2**, **4**, and **9**–**16** were inactive; ^b^ Compounds **2**, **4** and **9**–**16** were inactive (ED_50_ > 20 μg/mL); ^c^ (-): inactive; ^d^ Positive control: α-tocopherol. ^e^ Positive control: Mitomycin C.

**Figure 4:**
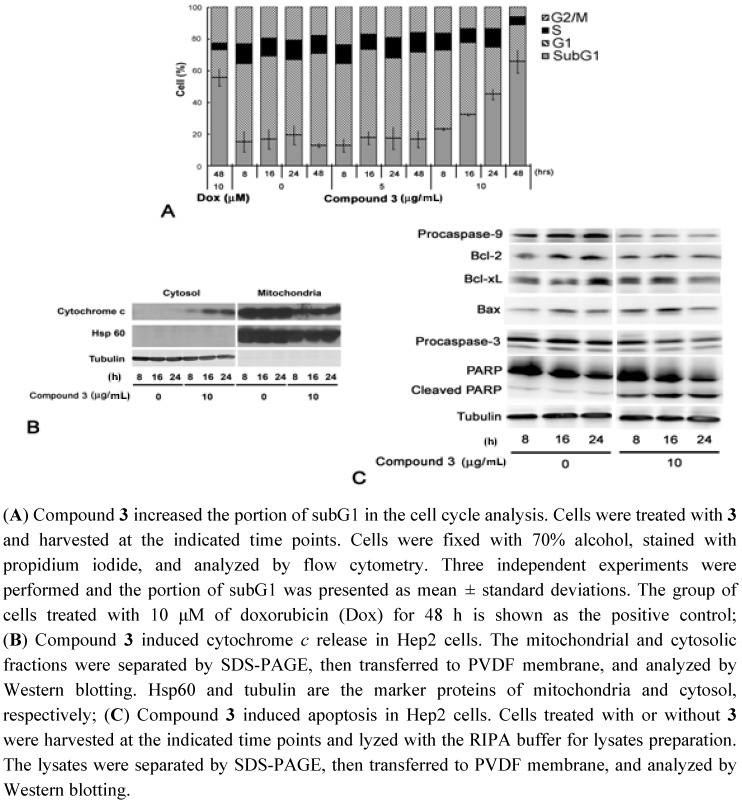
Mechanism of compound **3**-induced cell death.

### 2.3. Cell Cycle Analysis and Immunobloting

To investigate how **3** affects cell viability, we performed a cell cycle analysis. Hep2 cells treated with 10 μg/mL of **3** showed an elevating subG1 population (from 20% at 8 h to 60% at 48 h), but no difference in vehicle nor 5 μg/mL of **3** (Figure 4A). Compound **3**-induced apoptosis was determined by Western blotting. The cytosolic cytochrome c increased from 8 h to 24 h in the 10 μg/mL **3**-treated cells (Figure 4B). Furthermore, levels of procaspase-9, procaspase-3, and Bcl-2 were reduced in **3**-treated cells (Figure 4C). Then, caspase-3 mediated cleavage of PARP was also found (Figure 4C). Taken together, these results suggest that compound **3** triggers apoptosis through the intrinsic apoptosis pathway.

## 3. Experimental

### 3.1. General

The optical rotations were measured using a JASCO DIP-370 digital polarimeter. Infrared (IR) spectra were measured on a Mattson Genesis II spectrophotometer using a KBr matrix. UV spectra were measured a Hitachi U-3200 spectrophotometer. EI-MS were measured with JEOL Finnigan MAT TSQ-46C and JEOL SX-102A mass spectrometers. High-resolution mass spectra (HR-MS) were recorded by ESI MS on JEOL JMS-700 MStation and Hewlett-Packard 5989B mass spectrometers. ^1^H and ^13^C NMR spectra were obtained using Bruker Avance DRX 500 MHz and Bruker Avance 400 MHz spectrometers with tetramethylsilane (TMS) as the internal standard. Two-dimensional (2D) NMR experiments (HMQC, HMBC, and ROESY) were conducted using Bruker Avance DRX-500 and Bruker Avance 400 spectrometers. Sephadex LH-20 (Lipophilic Sephadex; Amersham Biosciences, Ltd.) and silica gel (230–400 mesh; Merck & Co., Inc.) were used for column chromatography, and pre-coated silica gel (60 F-254; Merck & Co., Inc.) plates were used for TLC. The spots on TLC were detected by spraying with 5% H_2_SO_4_ and then heating at 100 °C. Preparative HPLC was performed using a reverse phase column (Cosmosil 5SL-II column, 250 mm × 20 mm i.d.; Nacalai Tesque, Inc.) on a Shimadzu LC-6AD series apparatus with RID-10A refractive index and Prominence HPLC UV-Vis detectors.

### 3.2. Plant Material

The stems of *T. formosana* Nakai were collected in Ping-Tung County, Taiwan, in May, 2007. This material was identified by one of the authors (I.-S. Chen). A voucher specimen (code No. KTF200705A) has been deposited at the National Research Institute of Chinese Medicine, Taipei, Taiwan.

### 3.3. Extraction and Isolation

The dried stems of *T. formosana* Nakai (10.5 kg) were extracted two times with 95% EtOH (2 × 60 L). The EtOH extract was dried under reduced pressure at 45 °C to yield a brown syrup (*ca.* 650 g). The concentrated extract was subsequently partitioned successively with EtOAc (4 L × 4) and *n*-BuOH (4 L × 3) to afford 95 and 110 g, respectively. The EtOAc-soluble portion was subjected to flash chromatography on a silica gel column (60 × 300 mm), using CHCl_3_-MeOH (stepwise, 100:0 to 0:100, 10 L) to afford ten fractions (F-1 ~ F-10). Fraction 3 (F-3) was chromatographed over silica gel (30 × 200 mm) with *n*-hexane/EtOAc (40:1 ~ 0:1) to give four sub-fractions (F-3-1 ~ F-3-4). Sub-fraction (F-3-2) was purified over Sephadex LH-20 (20 × 900 mm) using CH_2_Cl_2_-MeOH (1:1, 500 mL) to produce six sub-fractions (F-3-2-1 ~ F-3-2-6). Sub-fraction (F-3-2-3) was purified by RP-HPLC (Cosmosil 5C^18^-AR-II column, 250 × 20 mm i.d.) using 65% CH_3_OH to yield **1** (16.5 mg) and **2** (12.8 mg). Fraction 9 (F-9) was chromatographed over silica gel (30 × 200 mm) with CH_2_Cl_2_/MeOH (50:1 ~ 0:1) to give ten sub-fractions (F-9-1 ~ F-9-10). Sub-fraction (F-9-1) was purified over Sephadex LH-20 (20 × 900 mm) using CH_2_Cl_2_-MeOH (1:1, 500 mL) to produce six sub-fractions (F-9-1-1 ~ F-9-1-6). Sub-fraction (F-9-1-5) was purified by RP-HPLC (Cosmosil 5C^18^-AR-II column, 250 × 20 mm i.d.) using 75% CH_3_OH to yield **3** (1.2 mg) and **4** (4.9 mg).

The *n*-butanol extract was further chromatographed on silica gel (40 × 300 mm) using an increasing gradient of MeOH in CH_2_Cl_2_ to obtain six fractions (F-1 ~ F-6). Fraction (F-4) was subjected to Sephadex LH-20 column (20 × 900 mm) chromatography eluted with MeOH (300 mL) to afford five sub-fractions (F-4-1 ~ F-4-5). Sub-fraction (F-4-1) was purified by preparative HPLC (Cosmosil 5C^18^-AR-II, 250 × 20 mm i.d., 65% MeOH) to give **5** (2.2 mg), **6** (3.1 mg), **7** (0.9 mg), **8** (0.6 mg) and **9** (5.6 mg). Sub-fraction (F-4-2) (3.72 g) was rechromatographed over a Sephadex LH-20 column (20 × 900 mm) using CH_2_Cl_2_-MeOH (1:4, 300 mL) to give four sub-fractions (F-4-2-1 ~ F-4-2-4). Sub-fraction (F-4-2-3) (1.02 g) was fractionated over a reversed phase preparative HPLC (Cosmosil 5C^18^-AR-II column, 250 × 20 mm i.d., 50% MeOH) to afford **10** (1.2 mg), and **13** (6.8 mg). Compounds **14** (8.1 mg), **15** (2.1 mg), and **16** (1.7 mg) were purified from sub-fraction (F-4-2-4) (1.02 g) by PTLC using CH_2_Cl_2_/MeOH (6:1). Sub-fraction (F-4-3) (340 mg) was chromatographed on Sephadex LH-20 (20 × 900 mm) using MeOH (400 mL) to yield four sub-fractions (F-4-3-1 ~ F-4-3-4). Employing a reversed phase preparative HPLC (Cosmosil 5C^18^-AR-II column, 250 × 20 mm i.d., 45% MeOH), **17** (3.2 mg) and **18** (28.3 mg) were obtained from sub-fraction (F-4-3-2) (50 mg). Sub-fraction (F-4-3-4) was further purified by RP-HPLC (Cosmosil 5C^18^-AR-II column, 250 × 20 mm i.d.) using 65% MeOH to furnish **11** (14.7 mg) and **12** (49.8 mg). The purity of the isolated compounds ranged from 97.0% to 99.5% as assessed by analytical HPLC (Cosmosil 5C^18^-AR-II column, 250 × 4.6 mm i.d., 45% MeOH, UV detection, 254 and 210 nm, flow rate: 1.0 mL/min). 

### 3.4. Spectral Data

*Turformosin A* (**1**)*:* colorless amorphous solid; [α]_D_^25^: +16.6 (*c* 0.2, MeOH); UV (MeOH) λ_max_ (log ε) 229 (4.25), 289 (4.13), 317 (4.30) nm; IR (neat) *v*_ma__x_ 3,398, 2,938, 2,839, 1,687, 1,631, 1,603, 1,508 cm^−1^; HR-ESI-MS *m/z* 723.2420 [M+Na]^+^ (calcd. for C_39_H_40_O_12_Na, 723.2417); ^1^H- and ^13^C-NMR data, see Table 1.

*Turformosinic acid *(**2**): colorless amorphous solid; [α]_D_^25^: +122.3 (*c* 0.1, MeOH); IR (neat) *v*_ma__x_ 3,384, 2,938, 2,868, 1,566, 1,550, 1,376, 1,048; HR-ESI-MS *m/z* 502.3290 [M]^+^ (calcd. for C_30_H_46_O_6_, 502.3294); ^1^H- and ^13^C-NMR data, see Table 2.

### 3.5. Cytotoxicity Assay

Cytotoxicity against Hep2 (human laryngeal carcinoma), WiDr (human colon adenocarcinoma), Daoy (human medulloblastoma), and MCF-7 (human breast adenocarcinoma) cells was measured using a 3-(4,5-dimethylthiazole-2-yl)-2,5-diphenyltetrazolium bromide (MTT) assay, based on reported methods [33]. Briefly, the cells were cultured in RPMI-1640 medium supplemented with serum in an atmosphere of 5% CO_2_ incubated at 37 °C. Test samples and the control drug standard were prepared at concentrations of 1, 10, 20, and 40 μg/mL. After seeding 2,880 cells/well in a 96-well microplate for 4 h, 20 μL of sample or standard agent was placed in each well and incubated at 37 °C for 3 days. Twenty μL of MTT were added, and incubation continued for 5 h. After removing the medium and adding DMSO (200 μL/well) into the microplate with shaking for 10 min, the formazan crystals (the product of MTT reacting with dehydrogenase existing in mitochondria) were re-dissolved, and their absorbance was measured on a model MR 7000 microtiter plate reader (Dynatech International Corporation, Edgewood, New York, NY, USA) at a wavelength of 550 nm. The ED_50_ was defined as the concentration of test sample resulting in 50% reduction of the absorbance found with the untreated cells.

### 3.6. DPPH Radical Scavenging Activity

The stable 1,1-diphenyl-2-picrylhydrazyl (DPPH) radical was used for the determination of free radical-scavenging activity of the extracts and compounds. Test compounds (**1**–**6**, **9**–**16**, and **18**) (120 μL) were added to 30 μL of DPPH (0.75 mM). After 30 min at room temperature, the absorbance was recorded at 520 nm. The experiment was repeated three times. Radical**-**scavenging activity (%) was calculated by the following formula: {[Ab − (A − As)]/Ab} × 100, where Ab is the absorbance without sample, A is the absorbance with compound and DPPH, and As is the absorbance with compound only [34]. If the activity of the test compound was more than 70%, the ED_50_ value was calculated.

### 3.7. Cell Cycle and DNA Content Analysis

Cells were seeded in a 6-cm dish and cultured overnight. Cells were incubated with fresh medium containing compound **3**. At the indicated time points, attached cells were trypsinized and combined with those in the supernatant. Cells were fixed with 70% EtOH at −20 °C and treated with 0.1% Triton X-100 for 30 min at room temperature. Then, cells were incubated with 50 μg/mL of RNase A and 50 μg/mL of propidium iodide (Sigma, St. Louis, MO, USA) in PBS at room temperature for 15 min. The relative proportions of cells in the G_1_, S, and G_2_/M cell-cycle phases were estimated by compartment analysis of DNA fluorescence using fluorescence-activated cell sorter (FACS) flow cytometry (Becton Dickinson, San Jose, CA, USA). Data were analyzed using the CellQuest software (Verity Software House Inc., Topsham, ME, USA).

### 3.8. Preparation of Mitochondrial and Cytosolic Fractions

After being cultured overnight, cells were incubated with fresh medium containing compound **2**. At the indicated time points, cells were trypsinized and washed twice with ice-cold PBS. Cells were resuspended in ice-cold extraction buffer [20 mM HEPES, pH 7.4, 10 mM KCl, 250 mM sucrose, 1.5 mM MgCl2, 1 mM EDTA, 1 mM EGTA, 1 mM dithiothreitol, 1× protease inhibitor cocktail (Merck)] and incubated for 10 min at 4 °C. Cells were homogenized with 15 strokes of a prechilled homogenizer, and the homogenates were sequentially centrifuged at 1,000 × g and 12,000 × g for 5 and 30 min, respectively, at 4 °C. The supernatant was collected as the cytosolic fraction. The pellet was re-suspended with ice-cold lysis buffer [20 mM Tris-HCl, pH 7.5, 150 mM NaCl, 1% NP-40, 1 mM dithiothreitol, 1× protease inhibitor cocktail (Merck)] and incubated for 20 min at 4 °C. The lysates were centrifuged at 15,000 × g for 5 min, and the supernatant was collected as the mitochondrial fraction.

### 3.9. Immunoblotting

Samples were separated by SDS-PAGE and transferred to PVDF sheets. Membranes were blocked with 5% non-fat milk dissolved in PBST buffer [137 mM NaCl, 3 mM KCl, 10 mM Na2HPO4, 2 mM KH2PO4, 0.1% (v/v) Tween-20] for 1 h and incubated with the primary antibody at 4 °C overnight. Then, membranes were washed thoroughly with PBST buffer and incubated for 1 h with the secondary antibody at room temperature. Immunoreactive protein was visualized using Immobilon western chemiluminescent HRP substrate according to the manufacturer’s protocol (Millipore, Billerica, MA, USA). Antibodies against Bcl-2, Bcl-xL, and Bax were purchased from Santa Cruz Biotechnology (Santa Cruz, CA, USA). Antibodies against caspase-9 and PARP were obtained from BD Pharmingen (San Diego, CA, USA). The anti-caspase-3 antibody was purchased from Millipore (Billerica, MA, USA). Horse radish peroxidase (HRP)-conjugated anti-mouse IgG and HRP-conjugated anti-rabbit IgG were obtained from Jackson ImmunoResearch, Inc. (West Grove, PA, USA).

## 4. Conclusions

The EtOH extract of *T. formosana* Nakai was partitioned successively with EtOAc and *n*-BuOH. The EtOAc portion was fractionated by column chromatography on Sephadex LH-20 and silica gel, as well as separated by HPLC, to afford one new phenylpropanoid, turformosin A (**1**), one new triterpenoid, turformosinic acid (**2**), and two known compounds, (−)-(7′*S*,8′*S*)-*threo*-carolignan X (**3**) and 2α,3β,19β-23-tetrahydroxy-olean-12-en-28-oic acid (**4**). From the *n*-BuOH layer, fourteen known compounds, methoxyhydroquinone-4-β-D-glucopyranoside (**5**), methoxyhydroquinone-1-β-D-gluco-pyranoside (**6**), 3,5-dimethoxybenzyl alcohol 4-*O*-β-D-glucopyranoside (**7**), 4-(3-hydroxy-1-propenyl)-2,6-dimethoxyphenyl β-D-glucopyranoside (**8**), d-*threo*-guaiacylglycerol-*O*-β-D-glucopyranoside (**9**), (7*S*,8*R*)-syringoylglycerol-7-*O*-β-D-glucopyranoside (**10**), 6-*O*-(*p*-hydroxybenzoyl)-D-glucose (**11**), d-glucose-6(4-hydroxy-3-methoxybenzoate) (**12**), dihydrophaseic acid 4′-*O*-β-D-glucopyranoside (**13**), 3′-*O*-methyl ellagic acid 4-*O*-β-D-xylopyranoside (**14**), 3-*O*-methylellagic acid 4-*O*-α-L-rhamnopyranoside (**15**), uridine (**16**), adenosine (**17**), and β-sitosteryl-3-*O*-β-D-glucopyranoside (**18**), were obtained. Their structures were determined based on extensive spectroscopic analyses, especially 2D NMR (^1^H-^1^H COSY, HMQC, HMBC, and NOESY) as well as HRMS. On the antioxidation screening effects on DPPH for the isolated compounds, compounds **1**, **3**, **5**, and **6** showed significant antioxidant effects. On the other hand, compound **3** exhibited most potent cytotoxicity against WiDr and Hep2 cancer cell lins with IC_50_ of 4.45 and 3.60 μg/mL, respectively. Compound **3** modulates intrinsic apoptotic pathway proteins in Hep2 cells, as shown by change expression of Bcl-2 family. In addition, compound **3** triggers caspase activation and also induces the release of cytochrome *c* and activation of caspase-9/caspase-3/PARP cascade. 
